# Supplementary material for: A UMLS-based spell checker for natural language processing in vaccine safety
Source: BMC Med Inform Decis Mak. 2007 Feb 12;7:3. doi: 10.1186/1472-6947-7-3 (PMC1805499; doi:10.1186/1472-6947-7-3)
Supplement: Additional file 13 — RAP application directory. Sets up directory for RAP (RDF application for PHP) source code [file 1472-6947-7-3-S13.gz › rap/doc/phpdoc/li_ontModel.html]

xml version="1.0" encoding="iso-8859-1"?


ontModel

Description
:   Class trees  
    Index of elements  
    Todo List

Classes
:   Individual
:   OntClass
:   OntModel
:   OntProperty
:   OntResource
:   OntVocabulary
:   OWLVocabulary
:   RdfsVocabulary

Files
:   Individual.php
:   OntClass.php
:   OntModel.php
:   OntModelP.php
:   OntProperty.php
:   OntResource.php
:   OntVocabulary.php
:   OWLVocabulary.php
:   RdfsVocabulary.php

phpDocumentor v 1.3.0RC3
